# Prioritising Infectious Disease Mapping

**DOI:** 10.1371/journal.pntd.0003756

**Published:** 2015-06-10

**Authors:** David M. Pigott, Rosalind E. Howes, Antoinette Wiebe, Katherine E. Battle, Nick Golding, Peter W. Gething, Scott F. Dowell, Tamer H. Farag, Andres J. Garcia, Ann M. Kimball, L. Kendall Krause, Craig H. Smith, Simon J. Brooker, Hmwe H. Kyu, Theo Vos, Christopher J. L. Murray, Catherine L. Moyes, Simon I. Hay

**Affiliations:** 1 Spatial Ecology & Epidemiology Group, Department of Zoology, University of Oxford, Oxford, United Kingdom; 2 Wellcome Trust Centre for Human Genetics, University of Oxford, Oxford, United Kingdom; 3 Bill & Melinda Gates Foundation, Seattle, Washington, United States of America; 4 London School of Hygiene & Tropical Medicine, London, United Kingdom; 5 Institute for Health Metrics and Evaluation, University of Washington, Seattle, Washington, United States of America; 6 Fogarty International Center, National Institutes of Health, Bethesda, Maryland, United States of America; National Institute of Parasitic Diseases China CDC, CHINA

## Abstract

**Background:**

Increasing volumes of data and computational capacity afford unprecedented opportunities to scale up infectious disease (ID) mapping for public health uses. Whilst a large number of IDs show global spatial variation, comprehensive knowledge of these geographic patterns is poor. Here we use an objective method to prioritise mapping efforts to begin to address the large deficit in global disease maps currently available.

**Methodology/Principal Findings:**

Automation of ID mapping requires bespoke methodological adjustments tailored to the epidemiological characteristics of different types of diseases. Diseases were therefore grouped into 33 clusters based upon taxonomic divisions and shared epidemiological characteristics. Disability-adjusted life years, derived from the Global Burden of Disease 2013 study, were used as a globally consistent metric of disease burden. A review of global health stakeholders, existing literature and national health priorities was undertaken to assess relative interest in the diseases. The clusters were ranked by combining both metrics, which identified 44 diseases of main concern within 15 principle clusters. Whilst malaria, HIV and tuberculosis were the highest priority due to their considerable burden, the high priority clusters were dominated by neglected tropical diseases and vector-borne parasites.

**Conclusions/Significance:**

A quantitative, easily-updated and flexible framework for prioritising diseases is presented here. The study identifies a possible future strategy for those diseases where significant knowledge gaps remain, as well as recognising those where global mapping programs have already made significant progress. For many conditions, potential shared epidemiological information has yet to be exploited.

## Author Summary

Maps have long been used to not only visualise, but also to inform infectious disease control efforts, identify and predict areas of greatest risk of specific diseases, and better understand the epidemiology of disease over various spatial scales. In spite of the utilities of such outputs, globally comprehensive maps have been produced for only a handful of infectious diseases. Due to limited resources, it is necessary to define a framework to prioritise which diseases to consider mapping globally. This paper outlines a framework which compares each disease’s global burden with its associated interest from the policy community in a data-driven manner which can be used to determine the relative priority of each condition. Malaria, HIV and TB are, unsurprisingly, ranked highest due to their considerable health burden, while the other priority diseases are dominated by neglected tropical diseases and vector-borne diseases. For some conditions, global mapping efforts are already in place, however, for many neglected conditions there still remains a need for high resolution spatial surveys.

## Introduction

Maps provide an essential evidence-base to support progress towards global health commitments [1]. For example, they provide important baseline estimates of disease limits [2–7], transmission [8–10] and clinical burden [11–14]; underpin surveillance systems and outbreak tracking [15,16]; help target resource allocation from the macro- [17,18] through the meso- [19–22] to the micro-scale [23]; and inform international travel guidelines [24–26]. Significant developments in mapping techniques have occurred over the last few decades, particularly through the use of species distribution models and model-based geostatistics [1,27]. Similarly, disease data has become more widespread and easier to share [28]. Despite these advances however, a recent review of 355 clinically-significant infectious diseases (IDs) indicated that of the 174 IDs for which an opportunity for mapping was identified, only 4% had been comprehensively mapped [1]. For many of these conditions, there is a significant shortfall between existing maps and what can be achieved with contemporary methods and datasets.

Traditional mapmaking has focussed on a vertical, single-species approach, requiring highly labour intensive, and therefore expensive, manual data identification and assembly [13,21,29–31]. The present era of open-access big data, high computational capacity, and rapid software development offers new opportunities for scaling-up the spatial mapping of IDs, primarily through the automation of data gathering and geopositioning but ultimately also to mapping. The Atlas of Baseline Risk Assessment for Infectious Disease (abbreviated ABRAID, as in, “to awake”) is a developing software platform designed to exploit this opportunity and has the ambition to produce continuously updated maps for 174 IDs globally [28]. Realising automation of data retrieval and positioning at this scale is a practically non-trivial but conceptually simple, logistic scaling exercise. In order to automate mapping for each ID so that it is continuously updated and improved as new information becomes available, the spatial inference methods used need to be tailored to each unique ID epidemiology [28]. In some cases this will require disease-specific methodological developments. This requires substantial investment, so an objective and systematic approach is required to determine the order in which IDs are to be mapped.

The first stage in this process is to organise all IDs using a schema based upon shared biological and epidemiological traits; for example, “the mosquito-borne arboviruses”. Such groups will likely have similar mapping requirements, enabling synergies in data collation, covariate selection, increased efficiency (*i*.*e*. in software development), and more robust validation of outputs [32,33]. We refer to these disease groups as “mapping clusters” and they form the basic architecture of the prioritisation process.

To rationally prioritise mapping of these conditions, the diseases within each mapping cluster were evaluated based upon their global burden (both morbidity and mortality), as well as the disease’s importance amongst public health stakeholders. Data inputs are quantitative in nature and reliant on either independently derived data or data sourced from entire communities rather than selected expert individuals. Therefore, this proposed framework is unaffected by much of the subjectivity associated with other prioritisation studies, and also provides a platform for rapidly incorporating changes to existing diseases, as well as emerging novel public health threats. The prioritisation exercise helps to guide the order in which diseases are mapped to best support public health priorities; we argue that all relevant diseases can and should eventually be mapped.

A comprehensive atlas of IDs is of central importance in providing geographical context to the understanding of tropical disease and global health [34–36]. Moreover, as the atlas becomes more complete the overlay of maps will provide opportunities for investigating patterns of global disease diversity [37,38] and the process of disease emergence [39].

## Methods

### Method summary

In order to generate disease prioritisation standards, diseases with shared taxonomy and transmission characteristics were grouped together to create clusters. Diseases within each cluster were evaluated based upon two factors reflecting their importance from a public health perspective: (a) the global burden of the disease and (b) the current public health focus on the condition. Both metrics were assessed simultaneously in order to rank the clusters, and specific diseases were then identified for prioritisation.

### Selection of diseases for mapping

This study aimed to be comprehensive in its scope of IDs. All diseases identified in a previous review as meriting mapping were included [1]. This earlier study categorised 355 diseases into five classes: Option 1, indicating that the disease was unsuitable for occurrence based mapping methods; Option 2, mapping the observed occurrence of the disease; Option 3, mapping the maximum potential range of the disease using knowledge of vector, intermediate host and reservoir species; Option 4, using niche mapping methods such as boosted regression trees; and Option 5, where sufficient data exist to allow for global maps of variation in prevalence of infection and/or disease. Option 1 diseases included those that showed no sustained spatial variation in occurrence (*i*.*e*. had a cosmopolitan distribution) and had insufficient evidence to allow for the global mapping of variation in prevalence using advanced statistical methods such as model-based geostatistics. In cases where such information does exist, these diseases were promoted to Option 5 status. Revisions to the *Hay et al*. *(2013)* paper have led to the inclusion of tuberculosis, ascariasis, trichuriasis and trachoma—all previously listed as Option 1—as Option 5 diseases. Further revisions included the exclusion of New and Old World Spotted Fever Rickettsiosis and New and Old World Phlebovirus because their constituent diseases were included. In addition, *Plasmodium knowlesi* was included due to the increasing appreciation of its significance to human health in Southeast Asia [40,41]. The new revised total of diseases that warrant mapping was therefore 176. Those diseases not considered for mapping due to Option 1 classification are outlined and justified in the Supporting Information.

### Creating disease clusters

Diseases were grouped into clusters based on characteristics relevant to spatial epidemiology. Diseases were placed in the same cluster if they had the potential to mutually reinforce each other in terms of data assembly, mapping requirements and cross-validation of data by comparison of outputs. Clustering classifications were therefore based on the key factors influencing the approach taken for mapping.

At the coarsest level, pathogens were grouped by agent type (virus/bacteria/fungus/other) and the larger agent groupings were split into specific phyla (*e*.*g*. Nematoda and Platyhelminths) [42]. These relatively coarse groups reflect fundamental differences in life histories and epidemiology as well as the most basic taxonomic divisions. Within these broad groupings, the mode of transmission was used to create the final disease clusters. This is an important factor when mapping IDs, as the mode of transmission has a large influence on which abiotic correlates are relevant to the mapping process. For instance, the transmission limits of vector-borne diseases are restricted in part by the environmental suitability for the vector species in question, thus diseases spread by similar vectors will share covariates [43]. Similarly, sexually-transmitted diseases are likely to share mapping methods linked to human distribution and behaviour, whilst pathogens spread by water contact would share common traits linked to the environment; these groupings can therefore be logically considered together within a mapping framework. The mode of transmission classifications are defined in the previous publication [1].

### Assessing disease burden

The burden of each disease was assessed using the disability-adjusted life year (DALY) estimates from the 2013 Global Burden of Disease Study (GBD 2013) [44,45]. DALYs quantify both morbidity and mortality attributed to each disease and therefore better capture the total impact of a disease than do clinical cases or mortality alone [44,45]. The GBD’s systematic approach across a wide spectrum of diseases provides an extremely valuable resource from which to compare the relative impact of diseases on human health.

Wherever possible, direct links were made between the GBD estimates and diseases in the mapping list. The GBD disease categories, which are based upon the International Classification of Diseases and Related Health Problems (ICD-10) [46], do not always specify particular infectious agents, but rather focus on the clinical symptoms of infection, or non-specified disease groups. These aggregated DALY estimates had to be split across the relevant causative diseases in the mapping list, therefore the *Hay et al*. *(2013)* study was reconciled with the ICD-10 codes and then GBD categories in order to disaggregate the broader classifications such as “other diarrheal diseases” and “other neglected tropical diseases”. The full process is outlined in the associated Supporting Information, S1 Text.

Overall, 11 of the 176 mapping diseases could not be reconciled to the GBD categories. Some were not considered due to having unknown pathogenic agents (*e*.*g*. tropical sprue) and others were very rare and fell into ICD-10 categories that were assigned over various groupings (*e*.*g*. pentastomiasis). These diseases were allocated a nominal DALY of 100; this value, while arbitrary, is low enough to avoid skewing the analysis. For each cluster, the total DALYs for all diseases was calculated and contributed to the final analysis.

### Assessing global health community interest

Of equal importance is the need to produce maps for those diseases where there is the greatest demand, whether from international organisations or from local public health authorities. Measuring this factor was achieved by surveying a representative subset of potential end-users, to identify which diseases have been prioritised by major public health stakeholders: state-funded public health agencies, private companies (*e*.*g*. vaccine developers), political bodies, non-governmental advocates and practitioners, as well as the scientific research community. For each disease, the final policy score was the sum of three component scores: public health, stakeholder interest, status as a notifiable disease, and h-index.

Cases from the different categories of public health stakeholders were included to capture the spectrum of interest groups (see S1 Text for full listing). Each organisation’s mission statement and project pages were reviewed to identify the diseases contained in their public health portfolio. Depending on the type of stakeholder, this would indicate that the organisation would, for example, dedicate funding and effort towards control of that disease, advocate for the disease to governments or public health agencies, or dedicate research funding to the disease. Each disease was allocated one point per stakeholder reporting an interest in it. An inclusive approach was followed, whereby diseases were considered to be of interest to a stakeholder, irrespective of any hierarchy within the agency’s prioritisation system.

Another point was allocated to diseases which were notifiable to national disease control agencies. In order to mitigate spatial bias in the notifiable disease listed by different agencies, a search for countries which had readily-accessible and clearly defined domestic policy relating to named pathogens was performed, and one country from each of the main GBD defined regions was selected: USA (High Income), Brazil (Latin America and the Caribbean), Zambia (sub-Saharan Africa), United Arab Emirates (North Africa and the Middle East), India (South Asia), Malaysia (South East Asia, East Asia and Oceania) and Croatia (Central and Eastern Europe and Central Asia). Interest in these diseases at a domestic level suggests that there will be interest in maps of these diseases, as demonstrated by the presence of subscription-only online databases of maps including GIDEON [47] and the rapid expansion of real-time maps to which physicians are encouraged to contribute [15,28].

Academic output, a proxy of funder agency awards, but also of high-quality data availability [1], was quantified based on the *h*-index of each disease [48], as reported by Scopus [49]. More commonly used to assess a scientist’s productivity and impact, the *h*-index is used here to quantify the level of active interest across the academic community in each disease [50]. The *h*-index is the number of published papers (referring to a particular disease) that have been cited by at least as many other papers. In other words, an *h*-index of 7 signifies that 7 published papers including that disease name have been cited at least 7 times. For each disease in turn, Scopus citation numbers were generated for all publications referring to the disease (document search for "Disease Name" in "Article Title, Abstract, Keywords"). This Scopus search generates a Citation Tracker file showing the number of citations to each publication referring to the "Disease Name". Diseases were then categorised according to their *h*-index. Those for which there was evidence of very high scientific output scored 2 (*h*-index >100), those with intermediate *h*-index (>50–100) scored 1.5, while diseases with *h*-index of <50 scored 1.

The diseases classified as Option 4 (use niche modelling methods) and Option 5 (model prevalence or incidence) have the most epidemiological data available and have the greatest potential to benefit from a dedicated mapping exercise, but also require the most resources. Option 2 and 3 diseases are data-poor and both require mapping of occurrence data only [1], and therefore are significantly less time-intensive to map, limited to more simplistic analyses, than those diseases categorised as Option 4 and 5. Option 3 disease mapping relates potential transmission limits to aspects of vector biology. In cases where Option 4 and 5 diseases also have the same vector, the Option 3 disease will be considered as part of mapping these complementary diseases; where this is not the case, a disease’s transmission limits can be assessed through a mixture of literature surveys and occurrence data overlap. Option 4 and 5 diseases within the disease clusters were therefore prioritised and for each cluster, the average policy score for the Option 4 and 5 diseases was calculated and contributed to the final analysis. These diseases should be the primary focus of future cartographic efforts as these require the most attention and bespoke inputs to be generated.

### Mapping prioritisation ranking of diseases

The final step in the process was to combine these assessments to produce a ranking of disease clusters and therefore recommend diseases to prioritise for mapping. Each cluster was plotted on a graph based on its total DALYs and the average policy priority of its Option 4 and 5 diseases. Option 2 and 3 diseases were included in the cluster DALY scores in order to reflect the relative importance that each cluster represented in terms of burden of disease. One cluster may consist of a large number of minor diseases which, as a collective grouping, represent a significant problem—by retaining the DALY score, this burden is reflected, With the policy priority score however, the opposite is the case; inclusion of multiple low scoring diseases would down-weight the cluster as a whole. In scenarios where clusters consist of a diverse grouping of pathogens, averaging policy score across all conditions misrepresents those with a high policy priority and therefore masks these diseases in comparison to clusters that only consist of those diseases with high policy priority scores.

Each cluster was then evaluated based upon its distance from a hypothetical cluster which had the highest DALYs (*i*.*e*. that of HIV) and the highest policy score (*i*.*e*. that of Malaria) relative to a line drawn from this cluster to the origin; those closer to this hypothetical cluster, along this axis, were prioritised higher. As a result, the relative influence of burden and policy priority could be considered both simultaneously and independently. Within each cluster, the diseases to be prioritised (*i*.*e*. Option 4 or 5) were then reported (Table 1). The code to replicate this methodology is freely available from: https://github.com/SEEG-Oxford/prioritisation.

**Table 1:** Clusters indicated as mapping priorities with their constituent diseases recommended for distribution modelling and current global mapping projects identified.

Cluster (main diseases to map / total diseases in cluster)	Diseases within cluster, to map	Total cluster DALYs	Average policy score	Current global mapping projects
1. Malaria (n = 3/5)	*Plasmodium falciparum*	65,493,135	11.8	MAP [13,29,40]; WHO [81]
	*P*. *knowlesi*			
	*P*. *vivax*			
2. HIV (n = 1/1)	HIV	69,480,661	11	GBD [51]; UNAIDS [82]
3. Tuberculosis (n = 1/1)	Tuberculosis	49,816,215	11	GBD [51]
4. Food/Water-borne (Bacteria) (n = 1/4)	Cholera	9,962,003	8	
5. Water-borne (Platyhelminth) (n = 3/7)	*Schistosoma haematobium*	3,062,843	7.7	GAHI [83]; Global NTD database [31]
	*S*. *japonicum*			
	*S*. *mansoni*			
6. Trypanosomiasis (n = 2/2)	African trypanosomiasis	728,564	7.5	WHO [68,84]
	American trypanosomiasis			
7. Filariasis (n = 3/3)	Bancroftian filariasis	2,022,099	6.2	GAHI [85]
	*Brugia malayi*			
	*B*. *timori*			
8. Soil Transmitted Helminths (n = 3/3)	Ascariasis	4,029,403	5.3	GAHI [5,14]
	Hookworm			
	Trichuriasis			
9. Leishmaniasis (n = 3/3)	Cutaneous leishmaniasis (Old World)	4,283,139	5.2	SEEG [8]
	Cutaneous leishmaniasis (New World)			
	Visceral leishmaniasis			
10. Unknown agent (n = 1/4)	Tropical sprue	3,609,400	4	
11. Picornaviridae (n = 1/1)	Polio	116,065	6	The Global Polio Eradication Initiative [86]
12. Food/Water-borne (Nematode) (n = 1/13)	Dracunculiasis	422,476	2.5	
13. Fly-borne (Nematode) (n = 2/5)	Loiasis	711,246	4.3	WHO and APOC [87] [88]
	Onchocerciasis			
14. Direct contact (Bacteria) (n = 4/6)	Anthrax	1,030,777	4	
	Brazilian purpuric fever			
	Leprosy			
	Trachoma			GAT [10,89]
15. Mosquito-borne (Virus) (n = 15/26)	Barmah Forest disease	4,219,569	2.6	
	California serogroup viruses			
	Chikungunya			
	Dengue			SEEG [2,12]
	Japanese encephalitis			
	Murray Valley encephalitis			
	Rift Valley fever			
	Rocio			
	Ross River virus			
	Sindbis			
	St. Louis encephalitis			
	Venezuelan equine encephalitis			
	Western equine encephalitis			
	West Nile fever			
	Yellow fever			

* Indicates default null value.

MAP—Malaria Atlas Project; WHO—World Health Organization; GBD—Global Burden of Disease; GAHI—Global Atlas of Helminth Infections; SEEG—Spatial Ecology and Epidemiology Group; APOC—African Programme for Onchocerciasis Control; GAT—Global Atlas of Trachoma

## Results

### Organization of the mapping clusters

**Fig 1:**
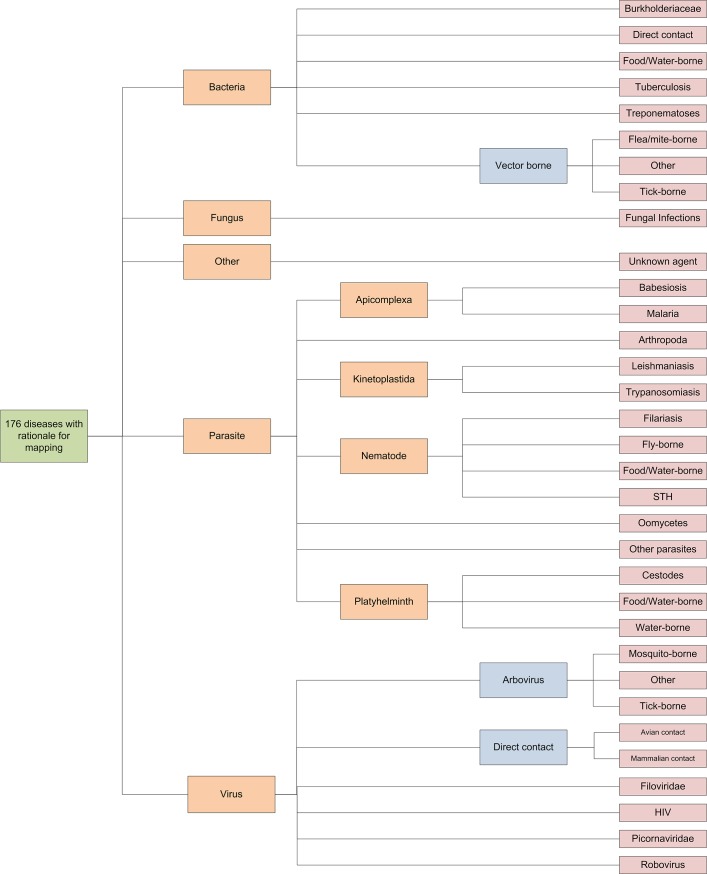
Hierarchical organisation of the 33 clusters. The 176 diseases with strong rationale for mapping were first sorted by taxonomy of pathogenic agent (in orange) and then structured by common epidemiological and transmission characteristics into sub-groupings (in blue) and finally clusters (in red). STH = soil transmitted helminth, VBD = vector borne disease.

The 176 diseases identified as having a rationale for mapping were organised into 33 clusters, based upon the biological and taxonomic classifications of the causative pathogen, modes of transmission and the mapping method recommended in a previous review [1] (Fig 1). Seven of these clusters included only a single disease due to their unique transmission within their broader taxonomic grouping (HIV, poliomyelitis, avian influenza, pythiosis, South American bartonellosis, tuberculosis and babesiosis). Conversely, the mosquito-borne arbovirus cluster was the largest cluster, consisting of 26 diseases, many of which have the potential to benefit from modelled maps.

### Prioritising mapping diseases

**Fig 2:**
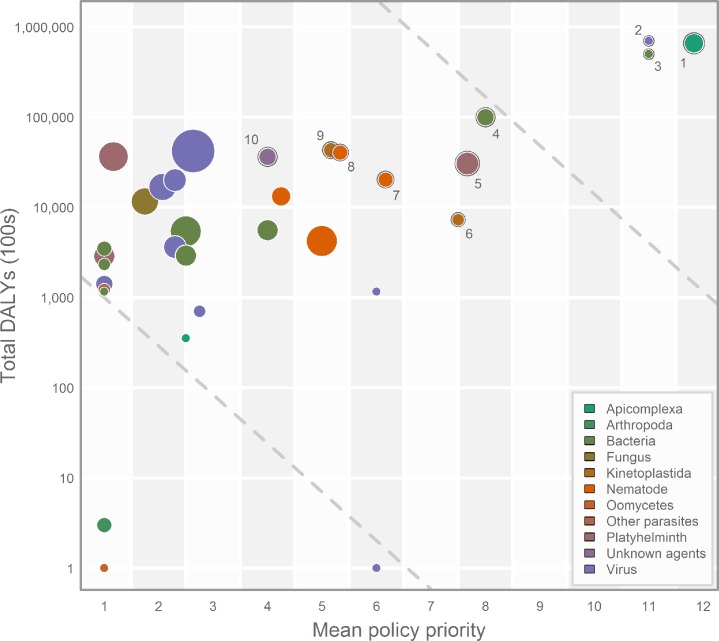
Disease prioritisation. Plot showing the 33 clusters of diseases as ranked by burden of disease DALYs (y-axis—logarithmic scale) and mean policy priority score of occurrence mapping and prevalence mapping diseases (x-axis—linear scale). The top ten clusters circled and numbered as identified in Table 1. The size of the circle is determined by the total number of diseases contained and colour is based upon taxonomy (as outlined by Fig 1; the web appendix contains the full disease listing for each cluster). The dashed guidelines are perpendicular to the axis along which prioritisation order for the clusters was determined; those closer to the top right, along this axis, were prioritised higher.

**Fig 3:**
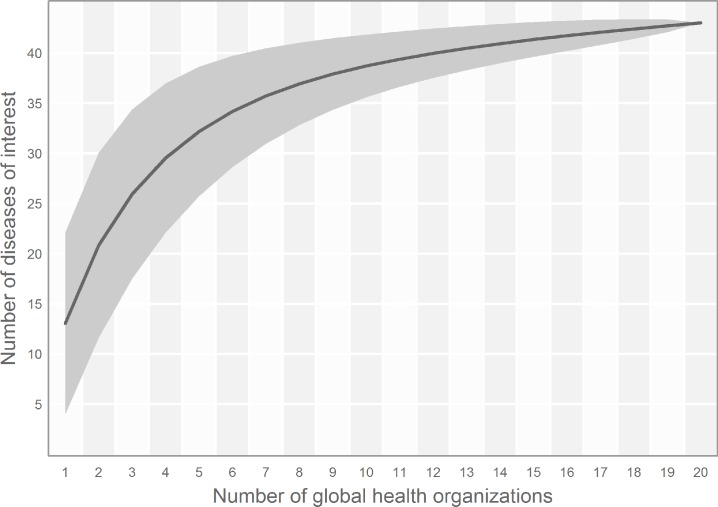
A “species accumulation” curve showing the cumulative number of diseases of interest sampled by increasing numbers of public health stakeholders examined. The diseases of interest of twenty global health stakeholders was indexed and plotted (see Methods). As additional organisations are sampled beyond the fifteen used in this study, the number of unique diseases identified plateaus at around 42. Thus not all public health stakeholders need to be sampled to capture the global diversity of diseases of public health interest.

Fig 2 brings together the two indices selected to prioritise diseases for mapping—disability adjusted life-year (DALY) burden and relative stakeholder interest. These plots demonstrate that the HIV, malaria and tuberculosis clusters are exceptional in representing an overwhelming share of DALY burden [51] and being of highest priority to the global health community with their placement in the top right quadrant of the graph. These three clusters contain five individual diseases that are a mapping priority, malaria (*Plasmodium falciparum*, *P*. *vivax*, and *P*. *knowlesi*), HIV and tuberculosis. Table 1 shows the top 15 disease clusters (*i*.*e*. those in the top right of Fig 2), representing 44 individual diseases, with their associated scores. Fig 3 demonstrates that there exists a group of approximately 45 diseases that are the collective focus of public health agencies. The 44 diseases prioritised by this study include all those diseases that represent a significant cartographic challenge (*i*.*e*. those diseases requiring either species distribution modelling approaches to produce occurrence maps or model-based geostatistics to produce prevalence maps, n = 33) identified by these public health agencies, save rabies and avian influenza. The clusters are ranked in order, whilst the diseases within each cluster are alphabetical and should be considered equal on the basis of this prioritisation.

**Fig 4:**
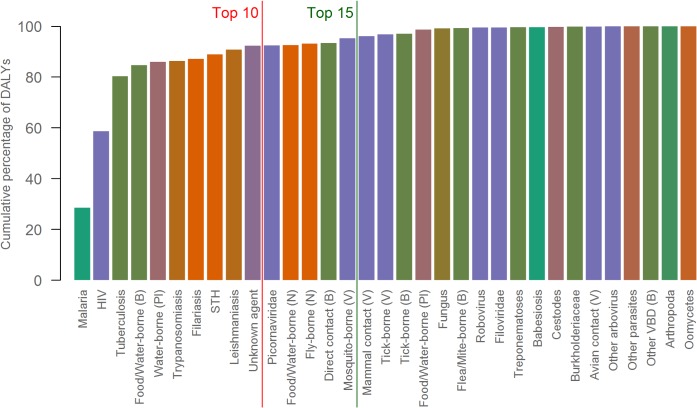
Cumulative percentage barplot indicating the cumulative percentage of DALYs accounted for by each cluster. The colouring is based upon taxonomy, as in Fig 2. The red line indicates the top ten clusters, the dark green indicates the top 15.

The top ten priority clusters account for over 92% of all DALYs for those IDs which require mapping (*i*.*e*. the 176 IDs identified); if this is expanded to the top 15 clusters containing 44 diseases to map, this value increases to 95% (Fig 4). Within these 44 diseases, 19 of the 29 neglected tropical diseases (NTD) highlighted by the WHO are represented. Within the top ten prioritised clusters, 14 individual diseases relate to these same NTDs [52,53]. The top 15 prioritised clusters include some diseases, such as the picornaviridae (polio), that have a low DALY burden but a high public health ranking because they are high on the eradication agenda.

### Disease burden

It was possible to establish a direct correspondence with GBD estimates for 34 of the 176 diseases with a strong rationale for mapping as listed by *Hay et al*. *(2013)* [44,45]. DALY estimates were allocated to a further 132 diseases by linking diseases with ICD-10 codes [46] and their respective GBD category definitions. Whilst these burden values are not accurate absolute values, and should not be interpreted as such, this DALY allocation does allow relative burdens to be determined. The remaining 11 diseases were given the baseline DALY allocation of 100, a value not intended to represent an estimate of the “true” DALYs associated with these diseases, but rather to distinguish them from diseases which were considered to cause a major burden in the GBD analysis. It is safe to assume that if such diseases were not assigned a specific GBD classification, their global impact on mortality and morbidity is relatively small.

**Fig 5:**
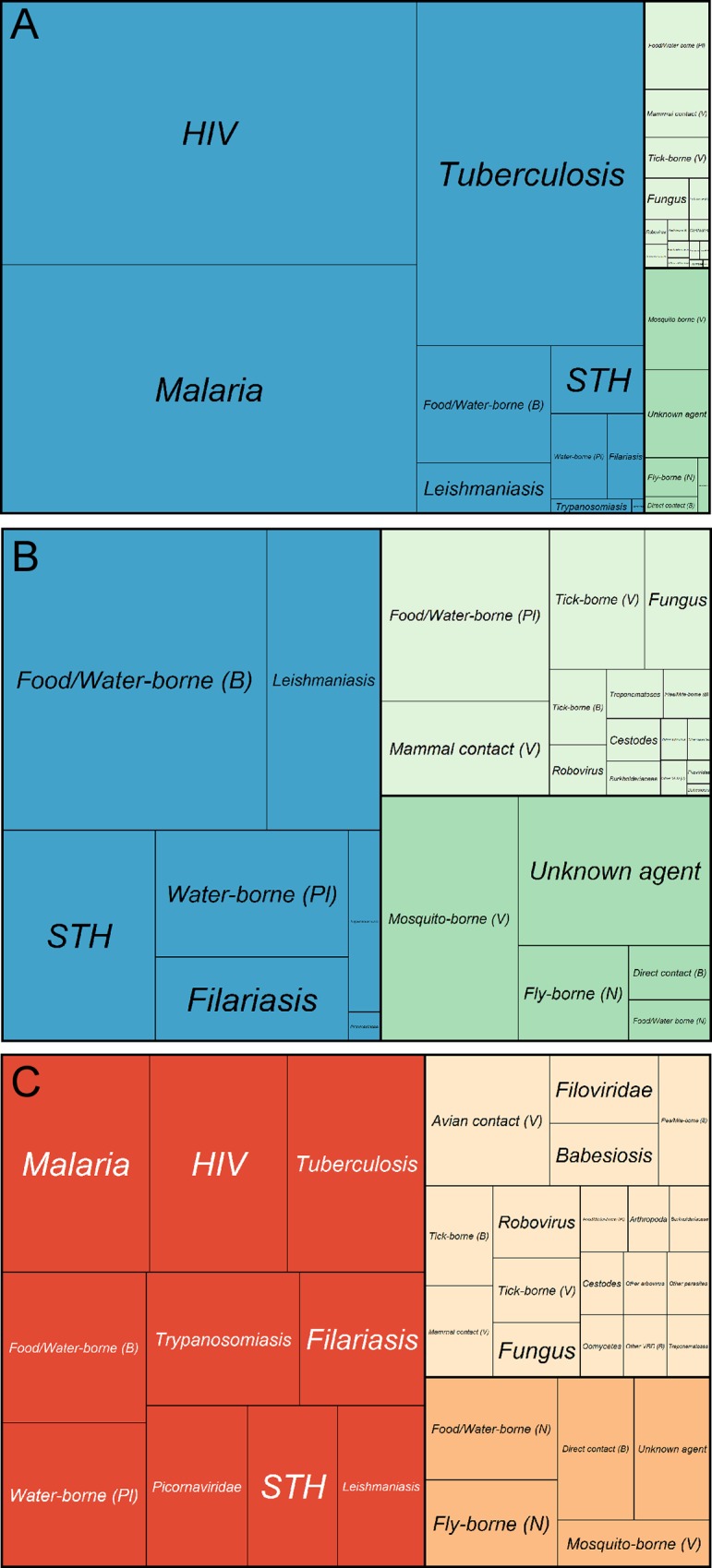
Plots indicating the relative importance of each mapping cluster. (A) Area of each section is determined by the total DALY contribution of each of the 33 clusters. Blue indicates a cluster contributing to the top ten clusters to be prioritised, green indicates top 44 diseases (n = 5 clusters) and light green represents the remaining disease clusters (n = 18). (B) Area of each section is determined by the total DALY contribution of 30 clusters, with HIV, tuberculosis and malaria excluded. Blue indicates a cluster contributing to the top ten clusters to be prioritised (n = 7), green indicates top 44 diseases (n = 5 clusters) and light green represents the remaining disease clusters (n = 18). STH = soil-transmitted helminth, (B)—bacteria, (N)—nematode, (Pl)—platyhelminth, (V)—virus. (C) Area of each section is determined by the total policy interest score of each of the 33 clusters. Red indicates a cluster within the top ten to be prioritised, orange indicates one of top 44 diseases (n = 5) and light pink represents the remaining disease clusters (n = 18). STH = soil-transmitted helminth, (B)—bacteria, (N)—nematode, (Pl)—platyhelminth, (V)—virus.

In total, the 176 diseases with a strong rationale for mapping [1] represent over 230 million DALYs, approximately 10% of the global DALY burden and 47% of the global ID DALY burden. At the cluster level, HIV, malaria and tuberculosis represent 80% of the overall mapping-disease DALY burden (Fig 5A). Apart from these three conditions, the only other IDs in the top 50 highest DALYs globally are not currently recommended for mapping because they do not show spatial variation in their occurrence and have insufficient data to map variation in disease prevalence with model-based geostatistical analyses. The high-burden diseases not currently considered for mapping include respiratory diseases, meningitis, and many diarrhoeal infections. Alternative approaches to mapping broader symptom groupings (severe pneumonia, severe diarrhoea and severe febrile illnesses) and then differentiating constituent disease components, are being developed. Together, this would map 80% of all DALYs caused by communicable diseases.

A higher resolution focus on the clusters excluding HIV, malaria and tuberculosis (Fig 5B) shows that over 60% of DALYs associated with the 176 IDs are accounted for by the other top ten prioritised clusters; approximately three quarters of the remaining DALYs are accounted for when the remaining prioritised clusters of diseases are included.

### Global health community interest

The treemap in Fig 5C displays the repartition of interest from the global health community across the clusters. Interest was scored in terms of: 1) the stated priorities of a survey of assorted public health stakeholders who are expected to be end-users of the maps, 2) status as a notifiable disease, and 3) prominence in the academic literature.

A total of 20 diverse stakeholders were surveyed. This was found to be a sufficiently large number to sample based on an analysis similar to a species accumulation curve that demonstrates the diminishing returns from increasing sampling effort [54]. The number of new diseases reported levelled off at around 15 organisations sampled (Fig 3) and so the 20 organisations used for this analysis was sufficient to capture the diseases of public health priority. Of the 176 diseases recommended for mapping [1], 24% were prioritised by at least one public health agency, and 55% were notifiable to at least one of the national disease control agencies.

Of those diseases that represent the greatest cartographic challenge, all were prioritised by at least one public health agency and two thirds were notifiable diseases. Of the 176 diseases, thirty diseases (17%) had an *h*-index [48] above 100 (with HIV having the highest *h*-index of 461), while 64% of the diseases had an *h*-index of 50 or less. Of the occurrence mapping and prevalence mapping diseases, 30% had an *h*-index above 100 and only 37% had an *h*-index of 50 or less.

Unlike the DALY burden, which was allocated at the disease level (S1 Text), the stated priority diseases were often grouped to the cluster level by the surveyed stakeholders. For instance, rather than specifying “*Plasmodium vivax*” or “visceral leishmaniasis” as a focus, “malaria” and “leishmaniasis” would be more commonly stated targets. Each component disease of these clusters would therefore be allocated a point, meaning that the number of component diseases in each cluster strongly inflated the overall interest score allocated at the cluster aggregate. Interest scores were calibrated in the final prioritisation assessment to the number of diseases classified as occurrence or prevalence mapping within each cluster (*i*.*e*. those requiring the more advanced geostatistical techniques, see Methods for more details), so as to avoid being unduly skewed by the size of the cluster.

Overall, malaria, HIV and tuberculosis were the leading clusters of interest, with scores of 11.8, 11 and 11, respectively. A further seven clusters received repeated interest, including food-borne/water-borne bacteria (score = 8) and water-borne trematodes (7.7), trypanosomiasis (7.5), filariasis (6.2), picornaviridae (6), avian contact viruses (6), soil-transmitted helminths (5.3) and leishmaniasis (5.2) all scoring highly, indicating their importance to the public health community. These scores are relative and intended to reveal general trends across the clusters rather than quantitatively reflect the weighting that any one institution places on a particular disease.

## Discussion

A review of all clinically significant IDs identified 176 with a strong rationale for mapping, of which only 4% have been adequately mapped [1]. The current study was undertaken to define a ruleset for determining which diseases, from a cartographic and public health perspective, should be prioritised when sequentially addressing this shortfall. Diseases were clustered together based upon shared characteristics (such as basic taxonomic division and mode of transmission) in order to consider together those diseases that would synergise operationally in terms of data collection, covariate selection and methodology used. Given the large number of diseases identified, prioritisation is necessary; we addressed this by evaluating both within the context of disease burden as well as considering the diseases’ influence within public health organisations and the wider academic community. It is important to stress that the study was focussed on priorities for mapping, and was not a general prioritisation of IDs; this is particularly important to emphasise given that a number of high-burden diseases, including meningitis, pneumonia and some diarrhoeal diseases, were not included in the list of 176 diseases [1,44,45].

Malaria is the infectious disease for which the most detailed and robust global risk maps exist [13,29]. The work of the Malaria Atlas Project [33,55] along with a proliferation of national and local-scale studies [56] has established a mature and sophisticated methodological approach centred on the use of model-based geostatistics to generate continuous surfaces of risk. This has been possible, in part, due to the long history of population-based malaria infection prevalence surveys where researchers and control programmes have used microscopy or rapid diagnostic tests to establish the proportion of randomly sampled individuals testing positive for malaria parasitaemia [30,57]. Crucial for geospatial mapping, such data are increasingly georeferenced with a latitude and longitude for each observation established *via* gazetteer methods (recorded location names linked to digital atlases) or directly using Global Positioning System (GPS) technology at the time of survey [58,59].

The high prioritisation of HIV and tuberculosis shown in the current study brings into sharp focus the need for similar mapping activities to be established for HIV and tuberculosis. All three diseases have an established history of routine and survey-based data collection that, in comparison to many other diseases, is of relatively high quality and consistency, laying the foundation for similar statistical mapping approaches to those used for malaria to be applied. A cornerstone of HIV surveillance over the last several decades has been routine blood testing for HIV infection in mothers attending sentinel antenatal clinics. Such data provide rich longitudinal observations of prevalence in this demographic group and the potential exists to combine these with cross-sectional data from nationally representative household surveys [60] to generate optimal space-time models of the changing geographical pattern of infection across individual countries. Unlike HIV and malaria, population-based tuberculosis prevalence testing is not currently included as part of the major international survey programmes [58,61]. However, such surveys (reporting on the prevalence of bacteriologically-confirmed pulmonary tuberculosis) have been undertaken in a number of high-burden countries in recent years, with many more planned in the near future [62]. In a similar way to HIV, the prospect exists of a mapping methodology that could combine survey-based data with the rich health-system based data on new case notifications and other metrics, leveraging the respective strengths of community- and facility-based data. A longer-term goal must be the development of a data assimilation and modelling architecture for all three of these major global diseases to support robust and regularly updated global maps detailing their joint distribution and its evolution though time which can be used to assess the impact of control and international financing efforts [18].

The current analysis identifies a number of different NTDs as priority diseases for mapping, a finding which is consistent with the emphasis given to mapping by the global NTD community in order to geographically target NTDs interventions [63,64]. Specifically, for those NTDs where morbidity control is the goal, including soil-transmitted helminths (STH) and schistosomiasis, interventions are most cost-effective when they are targeted to areas of highest transmission [21]. For those NTDs which are identified for elimination, such as onchocerciasis and lymphatic filariasis, it is essential to know where transmission occurs and when it has been successfully halted following control measures. As a consequence of these operational requirements, large-scale mapping initiatives are underway for each of the main NTDs (Table 1). A challenge for mapping the NTDs, and indeed for mapping many IDs, is the need to continually update maps in order to help track the progress in control. As interventions reduce transmission levels and therefore distributions become more focalised, the need for mapping will only increase.

Unsurprisingly, the top 44 diseases for prioritisation are dominated by those with the highest global burden. However, certain clusters stand out as having high public health attention without a high burden, particularly the picornaviridae cluster and its constituent disease, polio. Although cases are now restricted to a few hundred each year, polio has been identified as an eradication target and is a high priority for many public health stakeholders despite recent obstacles in the eradication schedule [65,66]. In these eradication and elimination scenarios, the role of mapping changes subtly to both identifying areas where cases continue to occur, and in highlighting potential future risks and improving surveillance [67]. Following a similar logic, diseases such as dracunculiasis, African trypanosomiasis and onchocerciasis, in spite of relatively low burdens, remain high policy priorities due to elimination efforts in various parts of the globe [68,69]. These examples demonstrate the utility of the approach used in this study of using assessments of the public health burden as well as metrics of public health attention.

The disease prioritisation methodology used here differs from existing approaches, such as the “Delphi panel method”, in that it does not include a panel of experts scoring various criteria associated with the diseases being considered [70–74]. In contrast, this study uses a simplified methodology, placing importance in reproducibility and flexibility, using clearly defined rules to assess available evidence and remove potentially subjective expert-opinion. The methods employed are reliant on independent, third party information, and are assessed in a consistent manner, which can easily respond to changes either in burden or public health focus. The relative importance of these diseases will most likely change over time, so an approach that can easily accommodate this is preferable. Burden estimation using the GBD is crucial, since it is the leading globally consistent measure by which to compare these various diseases and the effects of their many different clinical manifestations. Any global assessment of 301 causes of mortality and morbidity, and associated sequelae, will be subject to the limitations of data availability and epidemiological understanding as well as model assumptions and implementation [53,75,76], and will require frequent updates in a rapidly changing world. The technique presented here has the advantage of being rapidly updateable, and we will reproduce these numbers with each new iteration of the GBD project. As a consequence, public health authorities can also easily create bespoke prioritisation lists based upon a selection of disease inclusion criteria (such as those endemic to their particular country or region). This can more easily be achieved with the availability of sub-national estimates of disease burden from the GBD study. Country specific estimates of the interest scores can also be generated with greater specificity, and can therefore avoid some of the potential biases resulting from the use of other countries as representatives of each GBD region used in this study.

Additional factors that may influence the disease priority, such as potential economic impact [77–79], were not used in this analysis because insufficient information was available to include these metrics. The methodology outlined above benefits from two metrics that can be applied globally to quantify DALYs and public health priority. As and when measures of additional disease impacts become available, they can and should be incorporated into assessments such as this.

The study also identifies some high DALY groupings that do not have high-level policy interest. Three groupings (Tick-borne (Bacterial), Tick-borne (Viral) and Mammal contact (Viral)) have a cumulative high DALY burden, but relatively low policy rankings and therefore are just outside the top 15 cluster listing. This may reflect the large number of diverse pathogens that make up these groupings, many of which are relatively restricted in distribution and hence would not commonly be prioritised by globally focussed organisations. That said, the high DALY value indicates that these diseases are of international interest, particularly when secondary human-to-human transmission is a possibility such as with Lassa fever and Crimean Congo Haemorrhagic Fever [80]. These conditions further advocate the utility of regional and national level priority estimates.

The exclusion of diseases not suited for occurrence based mapping, and therefore omitted from the prioritisation process (so called Option 1 diseases [1]), is entirely based on cartographic considerations. Some of these diseases are inherently linked to human-to-human interactions, others are endogenous in origin, with the pathogen essentially ubiquitous amongst humans and only occasionally causing opportunist infections in certain scenarios, whilst some have the potential to cause infection anywhere across the globe due to the cosmopolitan distribution of their sources of infection, whether they be environmental or human based. Many of these diseases can vary spatially, as evidenced by the African meningitis belt, although such variation, when considered relative to the rest of the world, is due to differences in prevalence or intensity, not presence or absence. Occurrence based mapping methods, such as boosted regression trees, rely on binary presence/absence data. For conditions such as the common cold, diphtheria or respiratory syncytial virus, which have the potential to occur across the globe, these mapping techniques are ineffective. It is only through using more advanced methods, such as model-based geostatistics, that maps analysing the variation in intensity of these diseases can be produced. The limitation of this methodology is the amount of prevalence survey data required, which for many diseases is not comprehensive or detailed enough to allow for global analyses. Basic human related covariates, such as population density, urban extent profiles and national vaccination statistics can be used to explain a degree of the global variation in these diseases, but fall short of the wealth of information that can be derived from comprehensive global prevalence datasets, such as those available for malaria. As we continue to explore additional data avenues, there will be an increasing number of diseases where such data become available.

The disease prioritisation outlined in this study offers a logical framework for proceeding with disease mapping, which reinforces the necessity of existing programmes and identifies those diseases to focus on next (Table 1). Diseases which will form the initial focus of future study comprise both those with the highest-burden and those of greatest concern to the global health community. The initial top-priority diseases include a range of disease agents and transmission routes, and therefore present a variety of challenges for mapping. The prioritisation and clustering of these diseases presents a clear plan of action designed to maximise the effectiveness and value of future cartographic efforts.

## Supporting Information

S1 Text Supporting Information providing more specific details on the rationale for mapping, linking the Global Burden of Disease and those identified as mapping targets, feeds for the identifying diseases of interest to public health stakeholders and a full cluster ranking. (DOCX)
