# Supplementary material for: Prioritising Infectious Disease Mapping
Source: PLoS Negl Trop Dis. 2015 Jun 10;9(6):e0003756. doi: 10.1371/journal.pntd.0003756 (PMC4464526; doi:10.1371/journal.pntd.0003756)
Supplement: S1 Text — (DOCX) [file pntd.0003756.s001.docx]

**Supporting Information for “Prioritising infectious disease mapping”**

**Table: Diseases not recommended for mapping (Option 1) with an explanation for this classification (n=171) and potential alternative mapping opportunities.**

| **Disease** | **Reason for Option 1 (adapted from *Hay et al. (2013)*)** | **Other mapping opportunities and existing global outputs** |
| --- | --- | --- |
| Actinomycosis | Endogenous origin^1^ |  |
| Acute febrile respiratory disease, Adenoviral | Distribution tied to humans^2^ | Mapping of vaccine coverage |
| Adenovirus infection | Distribution tied to humans | Mapping of vaccine coverage |
| Adenoviral haemorrhagic conjunctivitis | Distribution tied to humans | Mapping of vaccine coverage |
| Amoeba – free living | Source of infection present worldwide |  |
| Amoebic abscess | Source of infection present worldwide |  |
| Amoebic colitis | Source of infection present worldwide |  |
| Animal bite-associated infection | Source of infection present worldwide |  |
| Anisakiasis | Source of infection present worldwide | Reported cases [[1](#_ENREF_1)] |
| Aspergillosis | Source of infection present worldwide |  |
| Bacillary angiomatosis | Source of infection present worldwide |  |
| Bacillus cereus food poisoning | Source of infection present worldwide |  |
| Bacterial vaginosis | Source of infection present worldwide |  |
| Bartonellosis – cat borne | Source of infection present worldwide | Reported outbreaks [[1](#_ENREF_1)] |
| Bartonellosis – other systemic | Source of infection present worldwide | Reported outbreaks [[1](#_ENREF_1)] |
| Blastocystis hominis infection | Source of infection present worldwide |  |
| Botulism | Source of infection present worldwide | Mapping of vaccine coverage; Reported outbreaks (using ProMED) [[1](#_ENREF_1)] |
| Brain abscess | Endogenous origin |  |
| Brucellosis | Source of infection present worldwide | National annual incidence levels [[2](#_ENREF_2)] |
| Campylobacteriosis | Source of infection present worldwide |  |
| Candidiasis (Yeast) | Endogenous origin |  |
| Chancroid | Distribution tied to humans |  |
| Chlamydia infections, misc. | Distribution tied to humans |  |
| Chlamydophila pneumonia infection | Source of infection present worldwide |  |
| Cholecystitis & cholangitis | Endogenous origin |  |
| Chronic fatigue syndrome | Unknown aetiological agent |  |
| Chronic meningococcemia | Source of infection present worldwide |  |
| Clostridial food poisoning | Source of infection present worldwide |  |
| Clostridial myonecrosis | Source of infection present worldwide | Mapping of vaccine coverage |
| Clostridium difficile colitis | Endogenous origin |  |
| Common cold | Distribution tied to humans |  |
| Conjunctivitis – viral | Distribution tied to humans | Mapping of vaccine coverage |
| Cryptococcosis (Yeast) | Source of infection present worldwide |  |
| Cryptosporidiosis | Source of infection present worldwide |  |
| Cutaneous larva migrans | Source of infection present worldwide |  |
| Cyclosporiasis | Source of infection present worldwide |  |
| Cysticercosis | Source of infection present worldwide | National level evidence of infection [[3](#_ENREF_3),[4](#_ENREF_4)] |
| Cytomegalovirus infection (Human herpesvirus 5) | Distribution tied to humans | Mapping of vaccine coverage; national estimates of seroprevalence in females [[5](#_ENREF_5)] |
| Dermatophytosis | Source of infection present worldwide |  |
| Dientamoeba fragilis infection | Source of infection present worldwide |  |
| Diphtheria | Distribution tied to humans | Mapping of vaccine coverage [[1](#_ENREF_1)]; national reported cases [[6](#_ENREF_6)] |
| Diphyllobothriasis | Source of infection present worldwide | Reported cases [[1](#_ENREF_1)] |
| Dipylidiasis | Source of infection present worldwide |  |
| Dirofilariasis | Source of infection present worldwide |  |
| Endocarditis – infectious | Endogenous origin |  |
| Enterobiasis | Source of infection present worldwide |  |
| Enteroviral hemorrhagic conjunctivitis | Source of infection present worldwide |  |
| Enterovirus infection | Source of infection present worldwide |  |
| Epidural abscess | Endogenous origin |  |
| Erysipelas or cellulitis | Source of infection present worldwide |  |
| Erysipeloid | Source of infection present worldwide |  |
| Erythrasma | Endogenous origin |  |
| Escherichia coli diarrhea | Source of infection present worldwide |  |
| Fungal infection – invasive | Endogenous origin |  |
| Gastroenteritis – viral | Source of infection present worldwide |  |
| Gianotti-Crosti syndrome | Unknown aetiological agent |  |
| Giardiasis | Source of infection present worldwide |  |
| Gonococcal infection | Distribution tied to humans |  |
| Granuloma inguinale (Donovanosis) | Distribution tied to humans | Reported cases [[1](#_ENREF_1)] |
| Hepatitis A | Source of infection present worldwide | Mapping of vaccine coverage; national estimates of seroprevalence [[7](#_ENREF_7)] |
| Hepatitis B | Distribution tied to humans | Mapping of vaccine coverage; national estimates of seroprevalence and distribution of genotypes [[8](#_ENREF_8)] |
| Hepatitis C | Distribution tied to humans | National estimates of seroprevalence [[9](#_ENREF_9)] and distribution of genotypes [[10](#_ENREF_10)] |
| Hepatitis D | Distribution tied to humans | National estimates of seroprevalence and distribution of genotypes [[11-13](#_ENREF_11)] |
| Hepatitis E | Source of infection present worldwide | Endemic status [[14](#_ENREF_14)] and distribution of genotypes [[15](#_ENREF_15)] |
| Hepatitis G | Distribution tied to humans |  |
| Herpes B infection | Source of infection present worldwide |  |
| Herpes simplex encephalitis | Distribution tied to humans |  |
| Herpes simplex infection | Distribution tied to humans |  |
| Herpes zoster | Source of infection present worldwide | Mapping of vaccine coverage |
| Hymenolepis diminuta infection | Source of infection present worldwide |  |
| Hymenolepis nana infection | Source of infection present worldwide |  |
| Infection of wound, puncture, IV line *etc*. | Distribution tied to humans |  |
| Infectious mononucleosis or EBV infection | Distribution tied to humans |  |
| Influenza | Source of infection present worldwide | Mapping of vaccine coverage |
| Intestinal spirochetosis | Endogenous origin |  |
| Intra-abdominal abscess | Endogenous origin |  |
| Intracranial venous thrombosis | Endogenous origin |  |
| Isosporiasis | Source of infection present worldwide |  |
| Kawasaki disease | Unknown aetiological agent |  |
| Keratoconjunctivitis, Adenoviral | Source of infection present worldwide |  |
| Kikuchi’s disease and Kimura disease | Unknown aetiological agent |  |
| Kingella infection | Endogenous origin |  |
| Laryngotracheobronchitis | Source of infection present worldwide |  |
| Legionellosis | Source of infection present worldwide |  |
| Leptospirosis | Source of infection present worldwide | Mapping of vaccine coverage; reported outbreaks [[1](#_ENREF_1)] |
| Listeriosis | Source of infection present worldwide | Reported outbreaks [[1](#_ENREF_1)] |
| Liver abscess – bacterial | Endogenous origin |  |
| Lymphocytic choriomeningitis | Source of infection present worldwide |  |
| Lymphogranuloma venereum | Distribution tied to humans |  |
| Malignant otitis externa | Endogenous origin |  |
| Measles | Source of infection present worldwide | Mapping of vaccine coverage; national reported cases [[6](#_ENREF_6)] |
| Meningitis – aseptic (viral) | Source of infection present worldwide | Mapping of vaccine coverage |
| Meningitis – bacterial | Source of infection present worldwide | Epidemic risk models [[16](#_ENREF_16)] |
| Microsporidiosis | Source of infection present worldwide |  |
| Moniliformis and Macaracanthorhynchus | Source of infection present worldwide |  |
| Mumps | Source of infection present worldwide | Mapping of vaccine coverage; national reported cases [[6](#_ENREF_6)] |
| Mycetoma | Source of infection present worldwide |  |
| Mycobacteriosis – *M. marinum* | Source of infection present worldwide |  |
| Mycobacteriosis – *M. scrofulaceum* | Source of infection present worldwide |  |
| Mycobacteriosis – miscellaneous nontuberculosis | Source of infection present worldwide |  |
| Mycoplasma (miscellaneous) infections | Distribution tied to humans |  |
| Mycoplasma pneumoniae infection | Source of infection present worldwide |  |
| Myiasis | Source of infection present worldwide |  |
| Necrotizing skin/soft tissue infections | Endogenous origin | Mapping of vaccine coverage |
| Orf | Source of infection present worldwide |  |
| Ornithosis | Source of infection present worldwide |  |
| Osteomyelitis | Endogenous origin |  |
| Otitis media | Endogenous origin | Mapping of vaccine coverage |
| Parainfluenza virus infection | Source of infection present worldwide |  |
| Parvovirus B19 infection | Source of infection present worldwide |  |
| Pediculosis | Source of infection present worldwide |  |
| Pericarditis – bacterial | Endogenous origin | Mapping of vaccine coverage |
| Perinephric abscess | Endogenous origin |  |
| Perirectal abscess | Endogenous origin |  |
| Peritonitis – bacterial | Endogenous origin |  |
| Pertussis | Source of infection present worldwide | Mapping of vaccine coverage [[1](#_ENREF_1)]; national reported cases [[6](#_ENREF_6)] |
| Pharyngeal and cervical space infections | Endogenous origin |  |
| Pharyngitis – bacterial | Source of infection present worldwide |  |
| Pityriasis rosea | Unknown aetiological agent |  |
| Plesiomonas infection | Source of infection present worldwide |  |
| Pleurodynia | Source of infection present worldwide |  |
| Pneumocystis pneumonia | Source of infection present worldwide |  |
| Pneumonia – bacterial | Endogenous origin | Mapping of vaccine coverage; national estimates of mortality [[17](#_ENREF_17)] |
| Protothecosis and chlorellosis | Source of infection present worldwide |  |
| Pseudocowpox | Source of infection present worldwide |  |
| Pyodermas (impetigo, abscess, *etc*.) | Endogenous origin |  |
| Pyomyositis | Distribution tied to humans |  |
| Q fever | Source of infection present worldwide | Mapping of vaccine coverage; reported outbreaks [[1](#_ENREF_1)] |
| Rat bite fever – spirillary | Source of infection present worldwide | Reported cases [[1](#_ENREF_1)] |
| Rat bite fever – streptobacillary | Source of infection present worldwide | Reported cases [[1](#_ENREF_1)] |
| Respiratory syncytial virus infection | Source of infection present worldwide | Mapping of vaccine coverage |
| Respiratory viruses – miscellaneous | Source of infection present worldwide |  |
| Reye’s syndrome | Unknown aetiological agent |  |
| Rheumatic fever | Source of infection present worldwide |  |
| Rhinoscleroma and ozena | Distribution tied to humans |  |
| Rhodococcus equi infection | Source of infection present worldwide |  |
| Roseola or human herpesvirus 6 | Distribution tied to humans |  |
| Rotavirus infection | Source of infection present worldwide | Mapping of vaccine coverage; national estimates of mortality [[18](#_ENREF_18)] |
| Rubella | Source of infection present worldwide | Mapping of vaccine coverage; national reported cases [[6](#_ENREF_6)] |
| Salmonellosis | Source of infection present worldwide |  |
| Sarcocystosis | Source of infection present worldwide |  |
| SARS | Transmission contained worldwide |  |
| Scabies | Source of infection present worldwide |  |
| Scarlet fever | Source of infection present worldwide |  |
| Septic arthritis | Endogenous origin |  |
| Septicemia – bacterial | Endogenous origin |  |
| Shigellosis | Source of infection present worldwide |  |
| Sinusitis | Distribution tied to humans | Mapping of vaccine coverage |
| Smallpox | Eradicated globally; present only in laboratory reserves |  |
| Sporotrichosis | Source of infection present worldwide |  |
| Staphylococcal food poisoning | Source of infection present worldwide |  |
| Staphylococcal scaled skin syndrome | Endogenous origin |  |
| Streptococcus suis infection | Source of infection present worldwide | Reported cases [[1](#_ENREF_1),[19](#_ENREF_19)] |
| Strongyloidiasis | Source of infection present worldwide |  |
| Subdural empyema | Endogenous origin | Mapping of vaccine coverage |
| Suppurative parotitis | Endogenous origin |  |
| Syphilis | Distribution tied to humans |  |
| Taeniasis | Source of infection present worldwide | National level evidence of infection [[3](#_ENREF_3)] |
| Tetanus | Source of infection present worldwide | Mapping of vaccine coverage [[1](#_ENREF_1)]; national reported cases [[6](#_ENREF_6)] |
| Thelaziasis | Source of infection present worldwide |  |
| Toxic shock syndrome | Endogenous origin | Mapping of vaccine coverage |
| Toxocariasis | Source of infection present worldwide | Mapping of vaccine coverage |
| Toxoplasmosis | Source of infection present worldwide | Mapping of vaccine coverage; seroprevalence status [[20](#_ENREF_20)] |
| Typhoid and enteric fever | Source of infection present worldwide | Mapping of vaccine coverage; national estimates of annual incidence [[21](#_ENREF_21),[22](#_ENREF_22)] |
| Typhus – endemic/murine (flea-borne) | Source of infection present worldwide |  |
| Urinary tract infection | Endogenous origin |  |
| Varicella | Distribution tied to humans | Mapping of vaccine coverage |
| Vibrio parahemolyticus infection | Source of infection present worldwide | Reported outbreaks [[23](#_ENREF_23)] |
| Whipple’s disease | Unknown transmission route |  |
| Yersiniosis | Source of infection present worldwide |  |
| Zygomycosis | Source of infection present worldwide |  |

1. Endogenous origin refers to infections caused by an agent already present on the body.
2. Distribution tied to humans refers to infections believed to be present wherever humans are.

**Allocating GBD estimates to all mapping diseases**

Quantified estimates allowing comparison of relative disease burden are an essential component to prioritising the diseases for mapping. The most contemporary and systematic estimates of disease burden are provided by the Global Burden of Disease Study (GBD) 2013, which calculates disability-adjusted life years (DALYs) for the major infectious, non-infectious and injury causes of death and morbidity globally [[24](#_ENREF_24),[25](#_ENREF_25)]. For consistency across the diseases, DALY numbers were exclusively taken from the GBD 2013 estimates.

GBD considered 67 categories or groupings relating to infectious diseases (including specific conditions such as “dengue” as well as larger groupings *e.g.* “Encephalitis”, “Food-borne trematodiases”, and also aggregated “Diarrheal diseases” categories), whilst the present study considered 176 infectious diseases identified for mapping. The process of appropriately allocating disease burden estimates across this larger number of diseases is described in this Appendix. It is worth specifying that many of the 67 infectious diseases were included in the Option 1 (*i.e.* diseases not currently recommended for mapping) category, due to being globally endemic. The table below summarises the 176 infectious diseases and their assigned DALYs. Further explanation is provided in the footnotes beneath.

Clusters were first classified by taxonomy of causative agent, then by similarities in transmission route [[26](#_ENREF_26)].

**Table outlining clusters, constituent diseases, Global Burden of Disease assignment and DALY score. (B) – bacteria, (N) – nematode, (Pl) – platyhelminth, (V) – virus.**

| Cluster | Disease name ^a^ | GBD assignment | DALYs assigned |
| --- | --- | --- | --- |
| Direct contact (B) | Anthrax | Other IDs | 116,065^1^ |
| Direct contact (B) | Brazilian purpuric fever | Other IDs | 116,065^1^ |
| Direct contact (B) | Leprosy | Leprosy | 39,707^2^ |
| Direct contact (B) | Mycobacteriosis – *M. ulcerans* | Other IDs | 116,065^1^ |
| Direct contact (B) | Trachoma | Trachoma | 171,170^2^ |
| Direct contact (B) | Tropical phagedenic ulcer | Not identified | 100^3^ |
| Food/Water-borne (B) | *Aeromonas* and marine *Vibrio* infections | Diarrheal diseases | 3,308,935^4^ |
| Food/Water-borne (B) | Cholera | Diarrheal diseases | 3,308,935^4^ |
| Food/Water-borne (B) | Enteritis necroticans | Diarrheal diseases | 3,308,935^4^ |
| Food/Water-borne (B) | Sennetsu neorickettsiosis | Other NTD | 35,198^5^ |
| Burkholderiaceae | Glanders | Other IDs | 116,065^1^ |
| Burkholderiaceae | Melioidosis | Other IDs | 116,065^1^ |
| Treponematoses | Endemic syphilis (bejel) | Other IDs | 116,065^1^ |
| Treponematoses | Pinta | Other IDs | 116,065^1^ |
| Treponematoses | Yaws | Other IDs | 116,065^1^ |
| Flea/Mite-borne (B) | Plague | Other IDs | 116,065^1^ |
| Flea/Mite-borne (B) | *Rickettsia felis* infection | Other NTD | 35,198^5^ |
| Flea/Mite-borne (B) | Rickettsialpox | Other NTD | 35,198^5^ |
| Flea/Mite-borne (B) | Typhus – epidemic | Other NTD | 35,198^5^ |
| Flea/Mite-borne (B) | Typhus – scrub (mite-borne) | Other NTD | 35,198^5^ |
| Flea/Mite-borne (B) | Relapsing fever | Other NTD | 35,198^5^ |
| Other VBD (B) | Bartonellosis – South American | Other IDs | 116,065^1^ |
| Tick-borne (B) | Tularemia | Other IDs | 116,065^1^ |
| Tick-borne (B) | Anaplasmosis | Other NTD | 35,198^5^ |
| Tick-borne (B) | Ehrlichiosis – human monocytic | Other NTD | 35,198^5^ |
| Tick-borne (B) | African tick bite fever | Other NTD | 35,198^5^ |
| Tick-borne (B) | Astrakhan fever | Other NTD | 35,198^5^ |
| Tick-borne (B) | Flinders Island spotted fever | Other NTD | 35,198^5^ |
| Tick-borne (B) | Israeli spotted fever | Other NTD | 35,198^5^ |
| Tick-borne (B) | Japanese spotted fever | Other NTD | 35,198^5^ |
| Tick-borne (B) | North Asian tick typhus | Other NTD | 35,198^5^ |
| Tick-borne (B) | Queensland tick typhus | Other NTD | 35,198^5^ |
| Tick-borne (B) | *Rickettsia sibirica mongolotimonae* infection | Other NTD | 35,198^5^ |
| Tick-borne (B) | Rocky Mountain spotted fever | Other NTD | 35,198^5^ |
| Tick-borne (B) | Lyme disease | Other NTD | 35,198^5^ |
| Tuberculosis | Tuberculosis | Tuberculosis | 49,816,215^2^ |
| Fungal infections | Chromomycosis | Other IDs | 116,065^1^ |
| Fungal infections | Entomophthoramycosis | Other IDs | 116,065^1^ |
| Fungal infections | Histoplasmosis – African | Other IDs | 116,065^1^ |
| Fungal infections | Lobomycosis | Other IDs | 116,065^1^ |
| Fungal infections | Penicilliosis | Other IDs | 116,065^1^ |
| Fungal infections | Rhinosporidiosis | Other IDs | 116,065^1^ |
| Fungal infections | Blastomycosis | Other IDs | 116,065^1^ |
| Fungal infections | Coccidioidomycosis | Other IDs | 116,065^1^ |
| Fungal infections | Histoplasmosis | Other IDs | 116,065^1^ |
| Fungal infections | Paracoccidioidomycosis | Other IDs | 116,065^1^ |
| Oomycetes | Pythiosis | Not identified | 100^3^ |
| Unknown agents | Brainerd diarrhoea | Diarrheal diseases | 3,308,935^4^ |
| Unknown agents | Tropical pulmonary eosinophilia | Not identified | 100^3^ |
| Unknown agents | Tropical sprue | Not identified | 100^3^ |
| Unknown agents | Viliuisk encephalomyelitis | Encephalitis | 300,265^6^ |
| Arthropoda | Pentastomiasis – *Armillifer* | Not identified | 100^3^ |
| Arthropoda | Pentastomiasis – *Linguatula* | Not identified | 100^3^ |
| Arthropoda | Tungiasis | Not identified | 100^3^ |
| Leishmaniasis | Leishmaniasis – cutaneous/mucosal, New World | Cutaneous leishmaniasis | 20,826^2^ |
| Leishmaniasis | Leishmaniasis – cutaneous/mucosal, Old World | Cutaneous leishmaniasis | 20,826^2^ |
| Leishmaniasis | Leishmaniasis – visceral | Visceral leishmaniasis | 4,241,487^2^ |
| Malaria | *Plasmodium knowlesi* | Malaria | 13,098,627^2^ |
| Malaria | *Plasmodium malariae* | Malaria | 13,098,627^2^ |
| Malaria | *Plasmodium ovale* | Malaria | 13,098,627^2^ |
| Malaria | *Plasmodium vivax* | Malaria | 13,098,627^2^ |
| Malaria | *Plasmodium falciparum* | Malaria | 13,098,627^2^ |
| Babesiosis | Babesiosis | Other NTD | 35,198^5^ |
| Trypanosomiasis | African trypanosomiasis | Trypanosomiasis | 390,075^2^ |
| Trypanosomiasis | American trypanosomiasis | Chagas | 338,489^2^ |
| Filariasis | Filariasis - Bancroftian | Lymphatic filariasis | 674,033^2^ |
| Filariasis | Filariasis – *Brugia malayi* | Lymphatic filariasis | 674,033^2^ |
| Filariasis | Filariasis – *Brugia timori* | Lymphatic filariasis | 674,033^2^ |
| Fly-borne (N) | Mansonelliasis – *M. ozzardi* | Other NTD | 35,198^5^ |
| Fly-borne (N) | Mansonelliasis – *M. perstans* | Other NTD | 35,198^5^ |
| Fly-borne (N) | Mansonelliasis – *M. streptocerca* | Other NTD | 35,198^5^ |
| Fly-borne (N) | Loiasis | Other NTD | 35,198^5^ |
| Fly-borne (N) | Onchocerciasis | Onchocerciasis | 1,179,826^2^ |
| Food/Water-borne (N) | Angiostrongyliasis | Other NTD | 35,198^5^ |
| Food/Water-borne (N) | Angiostrongyliasis – abdominal | Other NTD | 35,198^5^ |
| Food/Water-borne (N) | Baylisascariasis | Not identified | 100^3^ |
| Food/Water-borne (N) | Capillariasis – extra intestinal | Other NTD | 35,198^5^ |
| Food/Water-borne (N) | Capillariasis – intestinal | Other NTD | 35,198^5^ |
| Food/Water-borne (N) | Dioctophyme renalis infection | Other NTD | 35,198^5^ |
| Food/Water-borne (N) | Gnathostomiasis | Other NTD | 35,198^5^ |
| Food/Water-borne (N) | Gongylonemiasis | Other NTD | 35,198^5^ |
| Food/Water-borne (N) | Lagochilascariasis | Other NTD | 35,198^5^ |
| Food/Water-borne (N) | Mammomonogamiasis | Other NTD | 35,198^5^ |
| Food/Water-borne (N) | Oesophagostomiasis | Other NTD | 35,198^5^ |
| Food/Water-borne (N) | Trichostrongyliasis | Other NTD | 35,198^5^ |
| Food/Water-borne (N) | Dracunculiasis | Other NTD | 35,198^5^ |
| Soil-transmitted helminth | Ascariasis | Ascariasis | 1,271,708^2^ |
| Soil-transmitted helminth | Hookworm | Hookworm | 2,181,665^2^ |
| Soil-transmitted helminth | Trichuriasis | Trichuriasis | 576,030^2^ |
| Other parasites | Balantidiasis | Other intestinal infectious diseases | 61,179^8^ |
| Other parasites | *Entamoeba polecki* infection | Other intestinal infectious diseases | 61,179^8^ |
| Cestodes | *Bertiella* and *Inermicapsifer* | Other NTD | 35,198^5^ |
| Cestodes | Coenurosis | Other NTD | 35,198^5^ |
| Cestodes | Echinococcosis – American polycystic | Cystic echinococcosis | 60,557^2^ |
| Cestodes | Echinococcosis – mulitlocular | Cystic echinococcosis | 60,557^2^ |
| Cestodes | Echinococcosis - unilocular | Cystic echinococcosis | 60,557^2^ |
| Cestodes | Sparganosis | Other NTD | 35,198^5^ |
| Food/Water-borne (Pl) | Dicroceliasis | Food-borne trematodiases | 302,902^7^ |
| Food/Water-borne (Pl) | Echinostomiasis | Food-borne trematodiases | 302,902^7^ |
| Food/Water-borne (Pl) | Fasciolopsiasis | Food-borne trematodiases | 302,902^7^ |
| Food/Water-borne (Pl) | Gastrodiscoidiasis | Food-borne trematodiases | 302,902^7^ |
| Food/Water-borne (Pl) | Heterophyid infections | Food-borne trematodiases | 302,902^7^ |
| Food/Water-borne (Pl) | Metagonimiasis | Food-borne trematodiases | 302,902^7^ |
| Food/Water-borne (Pl) | Metorchiasis | Food-borne trematodiases | 302,902^7^ |
| Food/Water-borne (Pl) | Nanophyetiasis | Food-borne trematodiases | 302,902^7^ |
| Food/Water-borne (Pl) | Paragonimiasis | Food-borne trematodiases | 302,902^7^ |
| Food/Water-borne (Pl) | Clonorchiasis | Food-borne trematodiases | 302,902^7^ |
| Food/Water-borne (Pl) | Fascioliasis | Food-borne trematodiases | 302,902^7^ |
| Food/Water-borne (Pl) | Opisthorchiasis | Food-borne trematodiases | 302,902^7^ |
| Water-borne (Pl) | Cercarial dermatitis | Schistosomiasis | 437,549^2^ |
| Water-borne (Pl) | *Schistosoma haematobium* | Schistosomiasis | 437,549^2^ |
| Water-borne (Pl) | *Schistosoma intercalatum* | Schistosomiasis | 437,549^2^ |
| Water-borne (Pl) | *Schistosoma japonicum* | Schistosomiasis | 437,549^2^ |
| Water-borne (Pl) | *Schistosoma mansoni* | Schistosomiasis | 437,549^2^ |
| Water-borne (Pl) | *Schistosoma mattheei* | Schistosomiasis | 437,549^2^ |
| Water-borne (Pl) | *Schistosoma mekongi* | Schistosomiasis | 437,549^2^ |
| Mosquito-borne (V) | California serogroup viruses | Encephalitis | 300,265^6^ |
| Mosquito-borne (V) | Rift Valley fever | Other NTD | 35,198^5^ |
| Mosquito-borne (V) | Dengue | Dengue | 1,142,734^2^ |
| Mosquito-borne (V) | Ilheus and Bussuquara | Encephalitis | 300,265^6^ |
| Mosquito-borne (V) | Japanese encephalitis | Encephalitis | 300,265^6^ |
| Mosquito-borne (V) | Murray valley encephalitis | Encephalitis | 300,265^6^ |
| Mosquito-borne (V) | Rocio | Encephalitis | 300,265^6^ |
| Mosquito-borne (V) | Spondweni | Other NTD | 35,198^5^ |
| Mosquito-borne (V) | St. Louis encephalitis | Encephalitis | 300,265^6^ |
| Mosquito-borne (V) | Wesselsbron | Other NTD | 35,198^5^ |
| Mosquito-borne (V) | West Nile fever | Other NTD | 35,198^5^ |
| Mosquito-borne (V) | Yellow fever | Yellow fever | 30,680^2^ |
| Mosquito-borne (V) | Zika | Other NTD | 35,198^5^ |
| Mosquito-borne (V) | Coltiviruses – Old World | Other NTD | 35,198^5^ |
| Mosquito-borne (V) | Barmah Forest disease | Other NTD | 35,198^5^ |
| Mosquito-borne (V) | Chikungunya | Other NTD | 35,198^5^ |
| Mosquito-borne (V) | Eastern equine encephalitis | Encephalitis | 300,265^6^ |
| Mosquito-borne (V) | Karelian fever | Other NTD | 35,198^5^ |
| Mosquito-borne (V) | Mayaro | Other NTD | 35,198^5^ |
| Mosquito-borne (V) | Ockelbo disease | Other NTD | 35,198^5^ |
| Mosquito-borne (V) | O’nyong nyong | Other NTD | 35,198^5^ |
| Mosquito-borne (V) | Pogosta disease | Other NTD | 35,198^5^ |
| Mosquito-borne (V) | Ross River virus | Other IDs | 116,065^1^ |
| Mosquito-borne (V) | Sindbis | Other NTD | 35,198^5^ |
| Mosquito-borne (V) | Venezuelan equine encephalitis | Other NTD | 35,198^5^ |
| Mosquito-borne (V) | Western equine encephalitis | Encephalitis | 300,265^6^ |
| Other arbovirus | Bunyaviridae infections – miscellaneous | Other NTD | 35,198^5^ |
| Other arbovirus | Group C viral fevers | Other NTD | 35,198^5^ |
| Other arbovirus | Oropouche virus | Other NTD | 35,198^5^ |
| Other arbovirus | Sandfly fever | Other NTD | 35,198^5^ |
| Tick-borne (V) | Crimean-Congo hemorrhagic fever | Other NTD | 35,198^5^ |
| Tick-borne (V) | Alkhurma hemorrhagic fever | Other NTD | 35,198^5^ |
| Tick-borne (V) | Kyasanur Forest disease | Other NTD | 35,198^5^ |
| Tick-borne (V) | Louping ill | Encephalitis | 300,265^6^ |
| Tick-borne (V) | Omsk hemorrhagic fever | Other NTD | 35,198^5^ |
| Tick-borne (V) | Powassan | Encephalitis | 300,265^6^ |
| Tick-borne (V) | Tick-borne encephalitis | Encephalitis | 300,265^6^ |
| Tick-borne (V) | Tick-borne encephalitis - Russian spring summer | Encephalitis | 300,265^6^ |
| Tick-borne (V) | Thogoto | Encephalitis | 300,265^6^ |
| Tick-borne (V) | Colorado tick fever | Other NTD | 35,198^5^ |
| Avian contact (V) | Avian influenza | Not identified | 100^3^ |
| Mammal contact (V) | Chandipura and Vesicular stomatitis | Other NTD | 35,198^5^ |
| Mammal contact (V) | Hendra virus disease | Encephalitis | 300,265^6^ |
| Mammal contact (V) | Monkeypox | Other IDs | 116,065^1^ |
| Mammal contact (V) | Nipah and Nipah-like virus disease | Encephalitis | 300,265^6^ |
| Mammal contact (V) | Rabies | Rabies | 1,242,902^2^ |
| Mammal contact (V) | Tanapox virus disease | Not identified | 100^3^ |
| Mammal contact (V) | Vaccinia and cowpox | Not identified | 100^3^ |
| Filoviridae | Ebola | Other NTD | 35,198^5^ |
| Filoviridae | Marburg | Other NTD | 35,198^5^ |
| Picornaviridae | Poliomyelitis | Other IDs | 116,065^1^ |
| Robovirus | Argentine hemorrhagic fever (Junin virus) | Other NTD | 35,198^5^ |
| Robovirus | Bolivian hemorrhagic fever (Machupo virus) | Other NTD | 35,198^5^ |
| Robovirus | Brazilian hemorrhagic fever (Sabia virus) | Other NTD | 35,198^5^ |
| Robovirus | Lassa fever | Other NTD | 35,198^5^ |
| Robovirus | Venezuelan hemorrhagic fever | Other NTD | 35,198^5^ |
| Robovirus | Whitewater Arroyo virus infection | Other NTD | 35,198^5^ |
| Robovirus | Hantavirus infection – Old World | Other NTD | 35,198^5^ |
| Robovirus | Hantavirus pulmonary syndrome | Other IDs | 116,065^1^ |
| HIV | HIV | HIV | 69,480,661^2^ |

^a^ – for a more thorough description of pathogenic agents included in these terms, please refer to the publication by *Hay et al. (2013)*.

**Table footnotes**

1. **Other infectious diseases**

A variety of diseases are included in the GBD “Other infectious diseases” category (see appendix of GBD outputs for correspondence between GBD categorisations and ICD-10 codes [[24](#_ENREF_24),[25](#_ENREF_25),[27](#_ENREF_27)]). Of the 355 diseases listed in a previous study considering the diversity of infectious diseases [[26](#_ENREF_26)], 66 were classified as “Other infectious diseases”, each assigned 7,660,320/66 = 116,065 DALYs.

1. **Direct correspondences**

Diseases for which direct associations could be made between the nomenclature of the GBD and the list of mapping diseases are included in the table above. Some differences are apparent in the level of the nomenclature at the disease level, with GBD entries corresponding to clusters rather than individual diseases. For instance, the GBD “malaria” entry corresponds to the cluster level in the mapping list, which is made up of the five disease species (*Plasmodium falciparum*, *P. vivax, P.* *ovale, P. malariae* and *P. knowlesi*).

1. **Non-corresponding diseases**

For a handful of diseases, reconciliation of the disease with GBD (directly or through an ICD-10 code) was not possible. Typically these diseases were of endogenous origin (and were often classified as non-communicable within GBD) or related to ectoparasites. These diseases were each allocated a nominal 100 DALYs. Of the diseases included in the GBD, the infectious disease with lowest DALYs was yellow fever with 30,680, followed by leprosy with 39,707. The nominal DALY allocation to diseases not identified in the GBD list would therefore not skew any of the analytical outputs but enabled the diseases to be included in the analytical plots.

1. **Diarrhoeal diseases**

A variety of diseases are included in the GBD “Diarrheal diseases” category (see appendix of GBD outputs for correspondence between GBD categorisations and ICD-10 codes [[24](#_ENREF_24),[25](#_ENREF_25),[27](#_ENREF_27)]). Of the 355 diseases listed by *Hay et al. (2013)*, 22 were classified as “Diarrheal diseases”, each assigned 72,796,573/22 = 3,308,935 DALYs.

1. **Other neglected tropical diseases**

A variety of diseases are included in the GBD “Other neglected tropical diseases” category (see appendix of GBD outputs for correspondence between GBD categorisations and ICD-10 codes [[24](#_ENREF_24),[25](#_ENREF_25),[27](#_ENREF_27)]). Of the 355 diseases listed by *Hay et al. (2013)*, 89 were classified as “Other neglected tropical diseases”, each assigned 3,132,659/89 = 35,198 DALYs.

1. **Encephalitis**

A variety of diseases are included in the GBD “Encephalitis” category (see appendix of GBD outputs for correspondence between GBD categorisations and ICD-10 codes [[24](#_ENREF_24),[25](#_ENREF_25),[27](#_ENREF_27)]). Of the 355 diseases listed by *Hay et al. (2013)*, 16 were classified as “Encephalitis”, each assigned 4,804,232/16 = 300,265 DALYs.

1. **Food-borne trematodiases**

A variety of diseases are included in the GBD “Food-borne trematodiases” category (see appendix of GBD outputs for correspondence between GBD categorisations and ICD-10 codes [[24](#_ENREF_24),[25](#_ENREF_25),[27](#_ENREF_27)]). Of the 355 diseases listed by *Hay et al. (2013)*, 12 were classified as “Food-borne trematodiases”, each assigned 3,634,820/12 = 302,902 DALYs.

1. **Other intestinal infectious diseases**

A variety of diseases are included in the GBD “Other intestinal infectious diseases” category (see appendix of GBD outputs for correspondence between GBD categorisations and ICD-10 codes [[24](#_ENREF_24),[25](#_ENREF_25),[27](#_ENREF_27)]). Of the 355 diseases listed by *Hay et al. (2013)*, 7 were classified as “Other intestinal infectious diseases”, each assigned 428,255/7 = 61,179 DALYs.

**Ranking the disease-specific priorities of the global public health community.**

An important factor in determining a prioritization road-map for disease mapping is to ensure that diseases for which maps are in greatest demand are addressed first. This was achieved by a survey of a subset of anticipated end-users of the maps, to see which diseases were being targeted by the major public health stakeholders: publicly-funded health agencies, private companies (*e.g.* vaccine developers), political commitments, non-governmental organisation (NGOs), advocates and practitioners, as well as the scientific research community. Examples from the different categories of stakeholders were looked into to capture a sample across the spectrum of interest groups.

1. **International global health agencies**

Diseases of primary interest to the NGO and private sector of the global health community were surveyed from a varied spectrum of agencies. Agencies whose interests were focused on a single cause were excluded (*e.g.* President’s Malaria Initiative and the Polio Global Eradication Initiative); as were those whose scope was too comprehensive and far-reaching to allow determination of specific diseases which they prioritized (*e.g.*UNICEF, WHO and World Bank). The agencies included in the review are listed in the table below and were selected to cover a broad range of fields interested in public health. It was found that the diseases identified by such agencies reached a critical threshold of around 45 diseases – this number remained constant beyond approximately 15 agencies, as a result the specific agencies selected is not significant. Webpages describing each agency’s scope and priorities were searched, and all diseases cited as being a focus of the agency’s work were recorded. Diseases were allocated a point for each agency which targeted them.

**Tag-line descriptions and URLs of the fifteen agencies surveyed for their diseases of focus.**

| **Agency** | **Description (paraphrased from self-description)** | **URL** |
| --- | --- | --- |
| BMGF: Bill & Melinda Gates Foundation | Funding agency: harness advances in science and technology to save lives in developing countries. | http://www.gatesfoundation.org/What-We-Do |
| Carter Center | Focus on eradication and elimination of diseases. Health education and simple, low-cost methods. Surveillance and delivery systems. | http://www.cartercenter.org/health/index.html |
| CDC Neglected Parasite Infections | Focussed public health action focus on diseases selected based on: number of people infected, severity of the illness, preventability and treatability. | http://www.cdc.gov/parasites/npi.html |
| Clinton Foundation | Forms partnerships to strengthen health systems, increase access to lifesaving services, fight climate change, expand economic opportunities. | http://www.clintonfoundation.org/our-work/clinton-health-access-initiative |
| CORE Group | Brings together 70+ members to improve and expand community health practices. Supports NGOs and governments. | <http://www.coregroup.org/index.php> |
| GAVI | Increasing access to immunisation. | http://www.gavialliance.org/support/nvs/ |
| IVI | Private company: vaccine development | http://www.ivi.int/web/www/02_04 |
| London 2012 Declaration/WHO 2020 goals for NTDs | Commitment & collaboration by varied set of partners to address NTD disease burden. | http://unitingtocombatntds.org/ |
| MSF: Médecins Sans Frontières | Medical consultations: medical activities range from vaccination campaigns to complex surgery | http://www.msf.org.uk/medical-issues-0 |
| PATH | Driving transformative innovation to save lives. Development to delivery. | http://www.path.org/our-work/emerging-and-epidemic-diseases.php |
| PSI: Population Services International | Focus on serious challenges to global health. Communication & distribution efforts to ensure acceptance & proper use of health services & products. | http://www.psi.org/our-work/healthy-lives |
| Sabin | Private company: vaccine development. Development of sustainable, cost-effective vaccines. | http://www.sabin.org/programs/vaccine-development |
| Shantha Biotechnics | Private company: vaccine development. Develop cost-effective human health care products. | http://www.shanthabiotech.com/r&d.html |
| TDR: Special Programme for Research and Training in Tropical Diseases | Hosted by WHO, global programme for scientific collaboration to help facilitate, support and influence efforts to combat diseases of poverty. | http://www.who.int/tdr/diseases-topics/en/ |
| USAID | US government agency. Works to end extreme global poverty and enable resilient, democratic societies realise their potential. | http://www.usaid.gov/what-we-do/global-health |

1. **Notifiable Diseases**

Diseases which were notifiable to any of the following Public Health agencies were given one point. These countries were chosen in order to reflect the main GBD regions (‘High Income’, ‘Latin America and Caribbean’, ‘Sub-Saharan Africa’, ‘North Africa and Mediterranean’, ‘South Asia’, ‘Southeast Asia East Asia and Oceania’ and ‘Central and Eastern Europe and Central Asia’.

USA - <http://wwwn.cdc.gov/NNDSS/script/ConditionList.aspx?Type=0&Yr=2014>

Brazil - <http://www.anvisa.gov.br/hotsite/cruzeiros/documentos/2013/Annex%20II%20-%20%20NOTIFIABLE%20DISEASES%20IN%20BRAZIL.pdf>

Zambia – <http://www.hsa.org.za/misc/Notifiable%20Diseases.pdf>

United Arab Emirates - <https://www.haad.ae/HAAD/LinkClick.aspx?fileticket=NR4IhJoy5Bo%3D&tabid=1177>

India - <http://health.puducherry.gov.in/details_of_notifiable_diseases.htm>

Malaysia - [http://www.jknselangor.moh.gov.my/documents/pdf/sharingDoc/pdf/leptospirosis/Notifikasi.pdf](https://owa.nexus.ox.ac.uk/owa/redir.aspx?C=LYe87sozoE2Ll7VIj6l7Mg8OD86uBtIIvB1kxls8da2CnpqEE2Z8BmWA9E8-i0om-rxRv0M0Jnc.&URL=http%3a%2f%2fwww.jknselangor.moh.gov.my%2fdocuments%2fpdf%2fsharingDoc%2fpdf%2fleptospirosis%2fNotifikasi.pdf)

Croatia - <http://hzjz.hr/wp-content/uploads/2013/11/definicije_zb_12.pdf>

**Final cluster prioritisation ranking.** B = bacteria, Pl = platyhelminth, N = nematode, V = virus, VBD = vector-borne disease.

| **Ranking** | **Cluster** |
| --- | --- |
| 1 | Malaria |
| 2 | HIV |
| 3 | Tuberculosis |
| 4 | Food/Water-borne (B) |
| 5 | Water-borne (Pl) |
| 6 | Trypanosomiasis |
| 7 | Filariasis |
| 8 | Soil-transmitted helminths |
| 9 | Leishmaniasis |
| 10 | Unknown agent |
| 11 | Picornaviridae |
| 12 | Food/Water-borne (N) |
| 13 | Fly-borne (N) |
| 14 | Direct contact (B) |
| 15 | Mosquito-borne (V) |
| 16 | Mammal contact (V) |
| 17 | Tick-borne (V) |
| 18 | Tick-borne (B) |
| 19 | Food/Water-borne (Pl) |
| 20 | Fungus |
| 21 | Flea/Mite-borne (B) |
| 22 | Robovirus |
| 23 | Filoviridae |
| 24 | Treponematoses |
| 25 | Babesiosis |
| 26 | Cestodes |
| 27 | Burkholderiaceae |
| 28 | Avian contact (V) |
| 29 | Other arbovirus |
| 30 | Other parasites |
| 31 | Other VBD (B) |
| 32 | Arthropoda |
| 33 | Oomycetes |

**References**

1. Wertheim HFL, Horby P, Woodall JP (2012) Atlas of human infectious diseases (available online at: https://infectionatlas.org/). Oxford: Wiley-Blackwell. 280 p.

2. Pappas G, Papadimitriou P, Akritidis N, Christou L, Tsianos EV (2006) The new global map of human brucellosis. Lancet Infect Dis 6: 91-99.

3. Willingham AL, Engels D (2006) Control of *Taenia solium* cysticercosis/taeniosis. Adv Parasit 61: 509-566.

4. WHO Countries and areas at risk of cysticercosis, 2009. Available: <http://gamapserver.who.int/mapLibrary/Files/Maps/Global_cysticercosis_2009.png>. Accessed: July 2014

5. Cannon MJ, Schmid DS, Hyde TB (2010) Review of cytomegalovirus seroprevalence and demographic characteristics associated with infection. Rev Med Virol 20: 202-213.

6. WHO Immunization surveillance, assessment and monitoring: reported incidence time series. Available: <http://apps.who.int/immunization_monitoring/data/data_subject/en/index.html>. Accessed: July 2014

7. Jacobsen KH, Wiersma ST (2010) Hepatitis A virus seroprevalence by age and world region, 1990 and 2005. Vaccine 28: 6653-6657.

8. Kurbanov F, Tanaka Y, Mizokami M (2010) Geographical and genetic diversity of the human hepatitis B virus. Hepatol Res 40: 14-30.

9. Lavanchy D (2011) Evolving epidemiology of hepatitis C virus. Clin Microbiol Infect 17: 107-115.

10. Messina JP, Humphreys I, Flaxman A, Brown A, Cooke GS, et al. (2015) The global distribution and prevalence of HCV genotypes. Hepatology 61: 77-87.

11. Hughes SA, Wedemeyer H, Harrison PM (2011) Hepatitis delta virus. Lancet 378: 73-85.

12. Pascarella S, Negro F (2011) Hepatitis D virus: an update. Liver Int 31: 7-21.

13. Wedemeyer H, Manns MP (2010) Epidemiology, pathogenesis and management of hepatitis D: update and challenges ahead. Nat Rev Gastroenterol Hepatol 7: 31-40.

14. Goens SD, Perdue ML (2004) Hepatitis E viruses in humans and animals. Anim Health Res Rev 5: 145-156.

15. Purcell RH, Emerson SU (2008) Hepatitis E: An emerging awareness of an old disease. J Hepatol 48: 494-503.

16. Savory EC, Cuevas LE, Yassin MA, Hart CA, Molesworth AM, et al. (2006) Evaluation of the meningitis epidemics risk model in Africa. Epidemiol Infect 134: 1047-1051.

17. O'Brien KL, Wolfson LJ, Watt JP, Henkle E, Deloria-Knoll M, et al. (2009) Burden of disease caused by *Streptococcus pneumoniae* in children younger than 5 years: global estimates. Lancet 374: 893-902.

18. Glass RI, Parashar UD, Bresee JS, Turcios R, Fischer TK, et al. (2006) Rotavirus vaccines: current prospects and future challenges. Lancet 368: 323-332.

19. Lun ZR, Wang QP, Chen XG, Li AX, Zhu XQ (2007) *Streptococcus suis*: an emerging zoonotic pathogen. Lancet Infect Dis 7: 201-209.

20. Pappas G, Roussos N, Falagas ME (2009) Toxoplasmosis snapshots: Global status of *Toxoplasma gondii* seroprevalence and implications for pregnancy and congenital toxoplasmosis. Int J Parasitol 39: 1385-1394.

21. Connor BA, Schwartz E (2005) Typhoid and paratyphoid fever in travellers. Lancet Infect Dis 5: 623-628.

22. Crump JA, Luby SP, Mintz ED (2004) The global burden of typhoid fever. Bull World Health Organ 82: 346-353.

23. Nair GB, Ramamurthy T, Bhattacharya SK, Dutta B, Takeda Y, et al. (2007) Global dissemination of *Vibrio parahaemolyticus* serotype O3:K6 and its serovariants. Clin Microbiol Rev 20: 39-48.

24. GBD 2013 Disease and Injury Incidence and Prevalence Collaborators (2015) Global, regional, and national incidence, prevalence and YLDs for 301 acute and chronic diseases and injuries for 188 countries, 1990-2013: a systemtatic analysis for the Global Burden of Disease Study 2013. Under submission.

25. GBD 2013 Mortality and Causes of Death Collaborators (2015) Global, regional, and national age-sex specific all-cause and cause-specific mortality for 240 causes of death, 1990-2013: a systematics analysis for the Global Burden of Disease Study 2013. Lancet 385: 117-171.

26. Hay SI, Battle KE, Pigott DM, Smith DL, Moyes CL, et al. (2013) Global mapping of infectious disease. Philos Trans R Soc Lond B Biol Sci 368: 20120250.

27. WHO International statistical classification of diseases and related health problems 10th revision. Available: <http://apps.who.int/classifications/icd10/browse/2010/en>. Accessed: June 2014
